# Impact of Short-Term Exposure to Extreme Temperatures on Mortality: A Multi-City Study in Belgium

**DOI:** 10.3390/ijerph19073763

**Published:** 2022-03-22

**Authors:** Claire Demoury, Raf Aerts, Bram Vandeninden, Bert Van Schaeybroeck, Eva M. De Clercq

**Affiliations:** 1 Risk and Health Impact Assessment, Sciensano, 1050 Brussels, Belgium; raf.aerts@sciensano.be (R.A.); bram.vandeninden@sciensano.be (B.V.); eva.declercq@sciensano.be (E.M.D.C.); 2 Division Ecology, Evolution and Biodiversity Conservation, University of Leuven (KU Leuven), 3001 Leuven, Belgium; 3 Center for Environmental Sciences, University of Hasselt, 3590 Hasselt, Belgium; 4 Department of Meteorological Research and Development, Royal Meteorological Institute of Belgium, 1180 Brussels, Belgium; bertvs@meteo.be

**Keywords:** climate change, temperature, cause-specific mortality, case-crossover, DLNM, vulnerability, Belgium

## Abstract

In light of climate change, health risks are expected to be exacerbated by more frequent high temperatures and reduced by less frequent cold extremes. To assess the impact of different climate change scenarios, it is necessary to describe the current effects of temperature on health. A time-stratified case-crossover design fitted with conditional quasi-Poisson regressions and distributed lag non-linear models was applied to estimate specific temperature-mortality associations in nine urban agglomerations in Belgium, and a random-effect meta-analysis was conducted to pool the estimates. Based on 307,859 all-cause natural deaths, the mortality risk associated to low temperature was 1.32 (95% CI: 1.21–1.44) and 1.21 (95% CI: 1.08–1.36) for high temperature relative to the minimum mortality temperature (23.1 °C). Both cold and heat were associated with an increased risk of cardiovascular and respiratory mortality. We observed differences in risk by age category, and women were more vulnerable to heat than men. People living in the most built-up municipalities were at higher risk for heat. Air pollutants did not have a confounding effect. Evidence from this study helps to identify specific populations at risk and is important for current and future public health interventions and prevention strategies.

## 1. Introduction

According to the World Meteorological Organization (WMO), the past six years have been the warmest ever recorded [1] and global surface temperatures will continue to increase, as well as the frequency and intensity of hot extremes, in the future [2]. With global warming as well as significant excess mortality reported during hot extremes episodes worldwide [3,4,5], heat-related mortality has attracted attention and has been extensively investigated over the last decades [6,7]. Low-temperature extremes have been less studied, though they can have a strong impact on mortality [8,9]. For instance, cold-related mortality has been found to exceed heat-related mortality in recent global multi-country studies [10,11]. In Western Europe specifically, 140,271 deaths (125,698–153,056) were cold-related while 32,766 deaths (25,376–42,719) were due to non-optimum high temperatures [11]. The association between cold and mortality may also change with global warming and this is not well understood [12,13,14,15]. Most studies investigating the temperature–mortality relationship mainly focused on the effects of heat on all-cause mortality as in Belgium [16,17,18] where cold-related mortality nor cause-specific temperature–mortality relationships have been properly investigated.

Since the shape of the relationship between temperature and mortality can vary between areas [19], for instance within Europe [20] and even between nearby cities as in Belgium [18], it is important to investigate this association at the city and country levels but also to identify vulnerability factors depending on more local conditions such as urban characteristics (Sera et al., 2019) or specific individual characteristics of the population [21]. Investigations in regions with different environmental and climatic conditions contribute to the identification of risk factors to be addressed in the planning of public health interventions and prevention strategies [22]. Belgium has specific characteristics: it is a small densely populated country featuring a temperate maritime climate and a high degree of urbanization [23] prone to the urban heat island effect [24]. It records high levels of air pollution which may affect the link between temperature and mortality [25]. In the context of climate change, the investigation of the temperature–mortality association in Belgium would allow to better design public health measures towards populations with less adaptability to global warming [26].

The present study describes the effect of heat and cold extremes on mortality in nine Belgian urban agglomerations using daily maximum temperature and number of deaths for the period 2010–2015, accounting for the influence of humidity and air pollution levels. The association between all-cause and cause-specific mortality is investigated for different lag periods between the time of exposure and time of death, and for sub-populations stratified by both individual (sex, age) and municipality-level characteristics (socioeconomic status and level of built-up area).

## 2. Materials and Methods

### 2.1. Study Area

Belgium is situated in the Western Europe, has a maritime temperate climate (Köppen-Geiger type Cfb [27]) and a surface area of 30,688 km^2^. In 2015, the country counted 11,209,044 inhabitants spread over 589 municipalities with an averaged area of 52.0 km^2^ and a population count of 19,031 inhabitants. This study focuses on urban areas and includes 218 municipalities (52.9% of the total population, averaged area of 38.2 km^2^) which define the nine most-populated municipalities in Belgium and their agglomerations [28].

### 2.2. Data Collection

The daily number of deaths from natural causes was provided by Statbel, the Belgian statistical office, for the period from 1 January 2010 to 31 December 2015 including information on the municipality of residence at the time of death, sex, age (5-year age groups) and cause of death. We classified natural mortality according to the International Classification of Diseases, 10th revision (A00–R99) as well as cause-specific mortality from cardiovascular (I10–I70) and respiratory (J00–J99) diseases [29]. We further classified cardiovascular and respiratory diseases into subtypes: ischemic heart diseases (IHD) (I20–I25), cerebrovascular diseases (I60–I69), and chronic obstructive pulmonary diseases (COPD) (J40–J44, J47).

Daily meteorological data on maximum temperatures and relative humidity, averaged per municipality, were obtained from a 1 km resolution gridded dataset which results from the interpolation of quality-controlled station observations over Belgium [30,31].

For further analyses, we also collected municipality characteristics on the percentage of higher-education graduates as a marker of socioeconomic status [32]. We defined the built-up area of the municipalities according to their percentage of non-vegetated surface which was calculated with estimates developed from global training data derived using high-resolution imagery (MODIS/Terra Vegetation Continuous Fields, 250 m spatial resolution) [33]. Finally, daily averaged municipality concentrations (in µg/m^3^) of fine particulate matter (<2.5 micrometers in diameter, PM_2.5_), nitrogen dioxide (NO_2_), and ozone (O_3_) were calculated with data provided by the Belgian Interregional Environment Agency (RIO-IFDM model, at 100 m spatial resolution) [34].

### 2.3. Statistical Analyses

We used a time-stratified case-crossover design [35] to assess the association between temperature and mortality. In such a design, case days (days of death) are matched to control days selected from the same day of the week, month, and year as the case day. This design allows controlling for time-invariant confounders, including individual characteristics such as age and sex, and temporal confounders such as day of the week, seasonality, and time trends. We followed a two-stage approach. First, we performed conditional quasi-Poisson regressions, which allow accounting for over dispersion [36], with distributed lag non-linear models (DLNM) [37] to estimate the non-linear and delayed temperature–mortality associations in each agglomeration. In the second stage, the agglomeration-specific estimates were pooled using a random-effect meta-analysis [38] to describe the global association between temperature and mortality.

#### 2.3.1. Agglomeration-Specific Associations

In the first stage of the analysis, we used DLNM to describe the non-linear temperature–mortality and lag–mortality associations with spline functions. The agglomeration-specific cross-basis functions of daily mortality were modeled with a quadratic B-spline function for the temperature dimension with internal knots placed in equally spaced values; the lag dimension was modeled using a natural cubic spline with an intercept and internal knots placed in equally spaced values in the log scale. In line with similar studies [19,39,40], we extended the lag period to 21 days before death to exclude the harvesting effect; additionally, we considered other lag periods (0–7 days, 0–14 days, and 0–28 days) to fully capture complexities in temperature–mortality relationships. We defined a list of model candidates, specified by an increasing number of internal knots (*n* = 1, …, 5) for temperature and lag dimensions. The selected model was taken as the one featuring the minimal summed Q-AIC, a modification of the Akaike Information Criterion (AIC) for quasi-likelihood models [37], over all nine agglomerations [41]. We selected identical cross-basis functions for all agglomerations defined with knots placed at the same temperatures because we found agglomerations to have overlapping temperature ranges (see further Table 1). The estimated coefficients have thereby the same interpretation across agglomerations and their meta-analysis makes more sense. The quasi-Poisson regression model for each agglomeration assumes:

(1) log(E(Y))=cb+ns(humidity)+holiday+β×stratum

where *Y* is the daily counts of death; *cb* is the cross-basis function which defines the temperature–mortality and lag–mortality dimensions; *ns* is the natural cubic spline function with 3 degrees of freedom (df) of the daily mean relative humidity; *holiday* is a binary variable which allows to control for public holidays; and *stratum* allows to control for long-term and seasonal trends [36] and was defined as the same day of the week in the same month of the same year, and same municipality. We reduced the (three-dimensional) temperature– and lag–mortality associations to obtain overall cumulative (two-dimensional) temperature–mortality associations by cumulating the risks over the lag periods [38].

#### 2.3.2. Pooled Association

In the second stage of the analysis, we obtained the temperature–mortality association by pooling the agglomeration-specific estimates using a random-effect meta-analysis with restricted maximum likelihood estimation. The presence of heterogeneity between agglomerations was assessed using multilevel extensions of the Cochran Q test and I^2^ statistic [41]. We then derived the minimum mortality temperature (MMT) from the pooled overall cumulative association. We used the MMT as the reference for calculating the relative risks (RR) for temperatures corresponding to the 1st and 5th percentiles (cold effect) and 95th and 99th percentiles (heat effect) of the temperature distribution.

All analyses were performed in R (R Foundation for Statistical Computing, Vienna, Austria) using the packages gnm, dlnm, and mvmeta.

### 2.4. Cause-Specific Deaths and Subgroup Analyses

We performed stratification analyses by cause of death (cardiovascular, respiratory, IHD, COPC, cerebrovascular, other natural deaths), sex, age category (<65 years, 65–74 years, 75–84 years, and >85 years), and municipality characteristics, namely, education level and built-up area. For education, the municipalities were classified according to their percentage of higher-education graduates into low and high categories including 25% of the population (percentages of higher-education graduates <24.6% and >35.0%, respectively). Similarly, the municipalities were classified into low and high levels of built-up areas according to their percentage of non-vegetated surface (i.e., for percentages <18.1% and >30.5%, respectively).

### 2.5. Sensitivity Analyses

We conducted several sensitivity analyses to test the robustness of the results. We checked for the lag 1 and lag 3 of relative humidity (natural cubic spline function with 3 df) instead of the current day, and adjusted for the lag 1 and lag 3 of PM_2.5_, NO_2_ and O_3_ concentrations (linear functions). Furthermore, because exposure was assessed at the municipality of residence at the time of death, which can differ from the place of death, we performed a sub-analysis considering only the deaths for which the municipality of death was the same as the municipality of residence. Finally, we tested the robustness of our results to our modeling choices, i.e., the smoothness of the cross-basis definition function, by increasing by two the number of knots in the non-linear temperature– and lag–mortality associations from the DLNM.

## 3. Results

Over the period 2010–2015, there were 307,859 deaths from all causes in the studied municipalities (Table 1), including 91,327 (29.7%) deaths from cardiovascular diseases and 34,493 (11.2%) deaths from respiratory diseases (Table 2). The median of the daily maximum temperature was 15.4 °C and the nine agglomerations had largely overlapping temperature ranges (Table 1).

### 3.1. Agglomeration-Specific Associations

**Figure 1:**
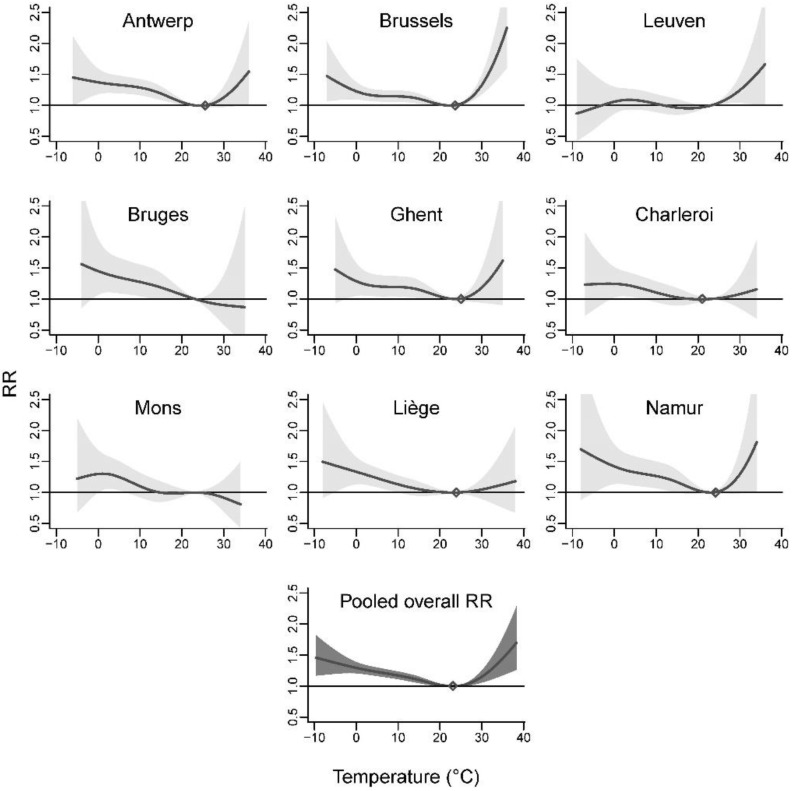
Agglomeration-specific and pooled overall temperature–mortality associations cumulated over lags of 0–21 days, 2010–2015.

The curves of the overall temperature–mortality associations cumulated over a lag of 0–21 days for the nine agglomerations showed differences but were generally U- or J-shaped (Figure 1). Agglomeration-specific MMT provided in Table 1 and Figure 1 (except for the agglomerations of Leuven, Bruges, and Mons whose curves did not show a minimum mortality value, see Figure 1) were similar in terms of percentile of the agglomeration-specific temperature distribution, at about 80th–90th percentiles for most agglomerations. In absolute value, the MMT varies from 21.0 °C (Charleroi) to 25.6 °C (Antwerp).

### 3.2. Pooled Association

When pooling the agglomeration-specific estimates using a random-effect meta-analysis, no heterogeneity was found (Cochran Q test: *p* = 0.37, I^2^ statistic: 5.8%). The risk decreased slowly for low temperatures, from extreme low to moderately low, and rose steeply for (extreme) high temperatures (Figure 1). Relative to the MMT (23.1 °C corresponding to the 86.3th percentile of the temperature distribution, Table 1), the RR for low temperatures at −1.7 °C (1st percentile) and 2.3 °C (5th percentile) were 1.32 (95% CI: 1.21–1.44) and 1.26 (95% CI: 1.19–1.34), respectively (Table 2). For high temperatures at 26.7 °C (95th percentile) and 31.3 °C (99th percentile), the RR were 1.04 (95% CI: 1.01–1.07) and 1.21 (95% CI: 1.08–1.36).

**Figure 2:**
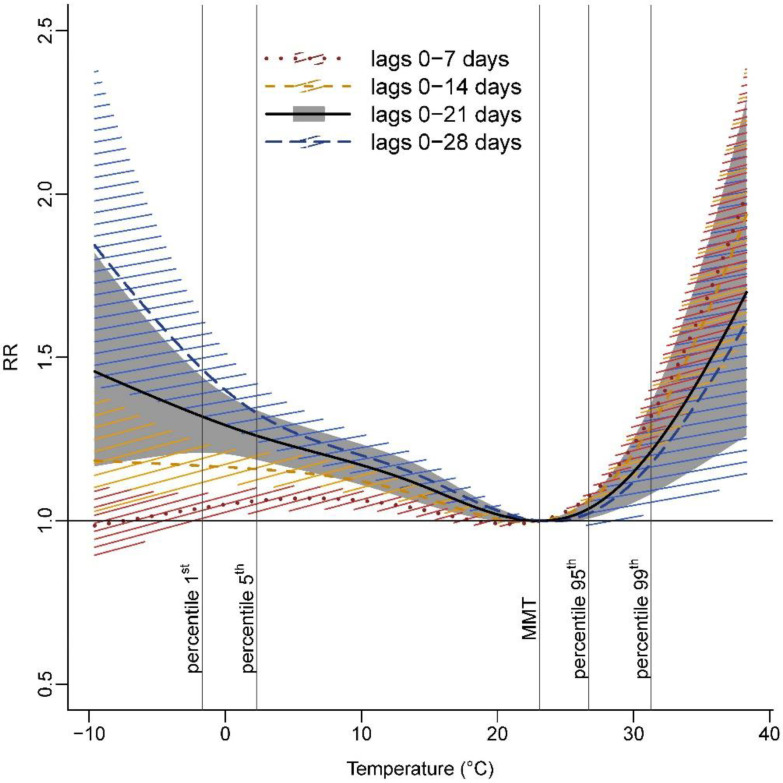
Pooled overall cumulative temperature–mortality associations for various lag periods, 2010–2015.

The pooled overall cumulative associations for lag periods of 0–7, 0–14, 0–21, and 0–28 days, described in Figure 2, showed shape differences (the corresponding agglomeration-specific relationships are shown in the Supplementary Materials, Figures S1–S3).

For enhanced visual comparability, temperature–mortality associations were centered on the MMT obtained from the pooled overall association for lags of 0–21 days (i.e., 23.1 °C). MMT for lag periods of 0–7, 0–14, and 0–28 days were, respectively, 21.0 °C, 22.1 °C, and 23.9 °C corresponding to the 78.0th, 82.5th, and 88.8th percentiles of the temperature distribution. Cumulating mortality risks over one week gave rise to enhanced heat effects while higher cold effects were found when cumulating mortality risks over four weeks (RR_p99, 0–7 days_ = 1.33, 95% CI: 1.24–1.43 and RR_p1, 0–28 days_ = 1.47, 95% CI: 1.32–1.63) (Figure 2, Supplementary Materials, Table S2).

### 3.3. Cause-Specific Deaths and Subgroup Analyses

**Figure 3:**
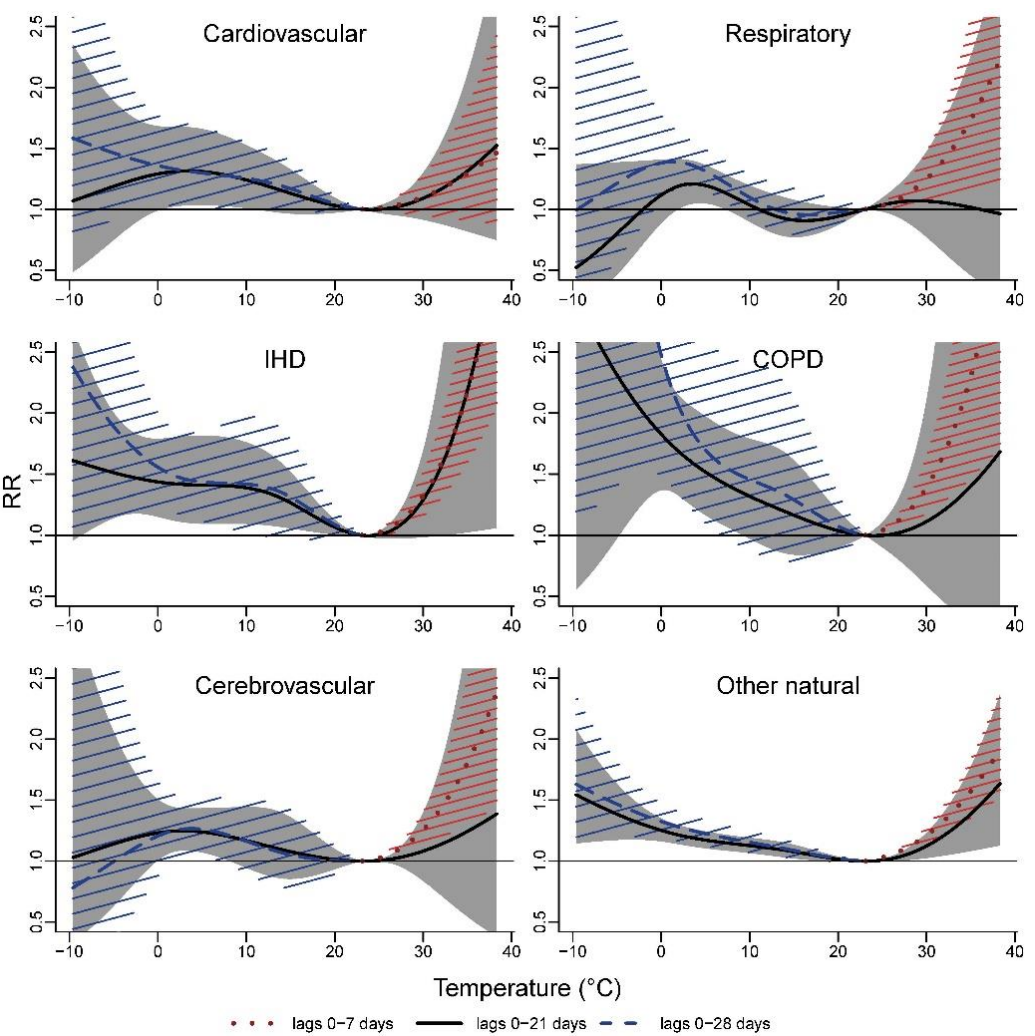
Pooled overall cumulative temperature–mortality associations by cause of death, 2010–2015.

In the cause-specific mortality investigation, despite the uncertainties observed for extreme temperatures, cold effects were found for all cause-specific mortality categories (Figure 3, for the lag periods of 0–7 and 0–28 days, only temperatures above and below the MMT, respectively, have been plotted). Cold effects were highest for IHD (RR_p5, 0–21 days_ = 1.42, 95% CI: 1.11–1.81) and COPD (RR_p5, 0–21 days_ = 1.66, 95% CI: 1.31–2.12) (Table 2). Effects of heat were observed in all groups when considering a risk cumulated over one week except for mortality from cardiovascular diseases which was borderline non-significant (Figure 3).

**Figure 4:**
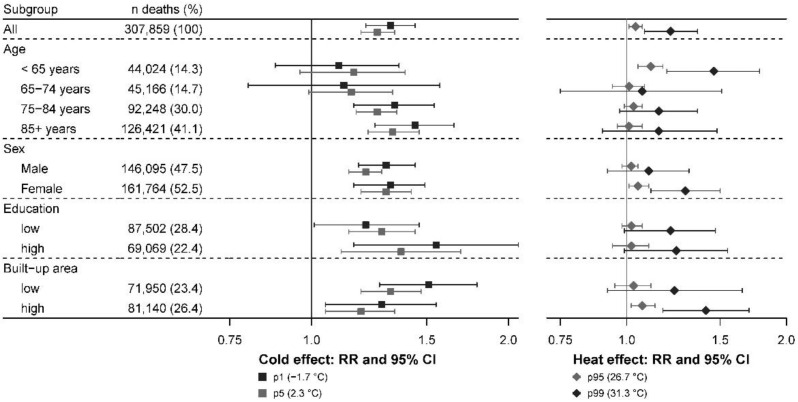
Cold effect (1st and 5th percentiles of temperature versus minimum-mortality temperature) and heat effect (95th and 99th percentiles of temperature versus minimum-mortality temperature) of temperature on mortality cumulated over lags of 0–21 days by subgroup, 2010–2015.

Figure 4 depicts the forest plots resulting from the subgroup analyses with a lag period of 0–21 days (the corresponding values can be found in the Supplementary Materials, Table S1). The results suggest higher cold effects in the groups of people aged 75–84 years and ≥85 years in comparison with younger people. For high temperatures, we found people aged ≥75 years being more vulnerable than people between 65 and 74 years. A higher risk for people aged <65 years was also observed. The results indicated stronger heat effects in women than in men. Because more women reached older ages (women represent 65.3% of the subjects ≥85 years in the study), at which time, people are more vulnerable to heat, we further stratified gender analyses by 5-year age groups and found higher risk estimates for women in the various age groups (Supplementary Materials, Figure S4). Of the 218 municipalities included in the study, 72 municipalities were classified into high education level areas where stronger cold effects were observed in comparison with the 49 municipalities classified with a lower level of education. Conversely, the results do not suggest differences in heat effects by the level of education. A lower cold effect and an indication for a stronger effect of heat were observed in the 28 municipalities with high levels of built-up area compared with the 148 municipalities with lower levels.

### 3.4. Sensitivity Analyses

In our sensitivity analyses, adjustment for air pollutants did not change the results to a considerable extent: cold effects were slightly increased when models included PM_2.5_ (lag 3), NO_2_, or O_3_ (Table 2). Heat effects were unchanged. Similarly, adjustment for lag 1 or lag 3 of the daily mean relative humidity slightly increased cold effects but left the heat effects unaffected (Supplementary Materials, Table S1). Restricting the analyses to the deaths for which the municipality of death was the same as the municipality of residence (Supplementary Materials, Table S1) caused a low magnitude reduction in cold (RR_p1, 0–21 days_ = 1.28, 95% CI: 1.12–1.46) and heat effects (RR_p99, 0–21 days_ = 1.15, 95% CI: 0.99–1.34). When increasing the degrees of freedom of the temperature– and lag–mortality relationships in the DLNM specification, the MMT obtained from the temperature–mortality associations showed higher values compared with the main results but cold and heat effects did not change considerably (Supplementary Materials, Table S2).

## 4. Discussion

This analysis provides evidence for significant associations between short-term risk of all-cause natural mortality and both low and high temperatures in Belgium, including cardiovascular and respiratory mortality. We identified differences in risk by age. A stronger vulnerability to heat was observed for women and for people living in the most built-up municipalities. The latter were, however, less vulnerable to low temperatures. Stronger cold effects were observed in highly-educated municipalities. Air pollutants did not have a considerable confounding effect.

In this pooled multi-city analysis for Belgium, we observed non-linear relationships between temperature and mortality, generally U- or J- shaped, with a slowly increasing risk with increasingly lower temperatures and a steeper increase for high temperatures, as it is generally observed [19,40]. Minimum mortality temperatures ranged from 21.0 °C to 23.9 °C (depending on whether the risk was cumulated over one or four weeks) which approximately correspond to the 80th and 90th percentiles of the exposure value distribution. These findings are consistent with studies performed in neighboring countries. Baccini et al. (2008) found a daily maximum temperature associated with the minimum mortality rate at 23.3 °C in Western Europe cities [6], 21.8 °C, 23.9 °C, and 24.1 °C in Zurich, London, and Paris, respectively. In a previous investigation carried out in Belgium in the cities of Antwerp and Brussels [18], minimum risk thresholds were observed at a daily maximum of 25.2 °C and 22.8 °C, respectively (considering a lagged average of 0–3 days) while Martinez et al. (2018) considered a minimum mortality at 26.0 °C for daily maximum temperature in Antwerp [17]. In the present study, the temperature–mortality relationships were separately investigated in nine agglomerations. Some agglomerations showed discrepancies with general findings, for instance, a decreasing risk with lower temperatures (Leuven) or higher temperatures (Bruges, Mons). This might be due to the uncertainties of the agglomeration-specific modeling due to the small number of deaths occurring on extremely cold or hot days. Although small numbers of deaths occurring on extreme days can result in high uncertainty in agglomeration-specific investigations, the pooled relationships show stable risk estimates.

In line with most studies [8,19], heat effects appear within a very short time after extremely hot days and do not last long. The underlying physiologic mechanisms with regard to heat-related mortality are associated with the thermoregulatory mechanisms of the body which put additional stress on the heart and lungs [42]. Cold effects appeared for moderate and extreme low temperatures, were more delayed than heat effects, and lasted for a few weeks. Physiological and pathological mechanisms on the association between low temperatures and cardiovascular mortality have also been postulated. Low temperatures have, for instance, been linked to increases in blood pressure and viscosity, platelet and red blood cell count [43], which may increase cardiovascular risks.

Although examining specific mortality causes led to smaller numbers and more imprecise estimates, our findings showed an association between temperature and cardiovascular and respiratory mortality. These associations seemed to be driven by the strongest risks found for ischemic heart diseases and chronic obstructive pulmonary diseases, which is consistent with other studies [39,40]. The present study suggested differences in vulnerability by age, sex, education, and urban characteristics. In line with the literature [44,45], we observed an increasing risk with both low and high temperatures as age progresses from 75 years onwards. Vulnerability for the elderly may be due to a limited thermoregulatory response, increased prevalence of comorbidities, a solitary life, reduced access to air conditioning or heating systems, bed confinement, poor self-care, or difficulties in accessing healthcare facilities [22,44,46]. Compared with the elderly, we found a greater risk of death associated with high temperatures for people aged less than 65 years. These findings differ from the current evidence [21,47] but may be partly explained by the inclusion of children, especially children under the age of one, who are particularly vulnerable to heat [42,48]. Since adults aged below 65 years are not considered as a vulnerable group to heat [22], this might also be explained by a lack of interventions to protect this specific group. Women were more at risk than men with respect to heat. This result may be due to differences in physiology or exposure patterns between men and women [22]. People of lower socioeconomic status are generally observed to be more vulnerable to heat than groups with higher socioeconomic status [21,47,49]. Education may, for example, influence the understanding of preventive messages. By contrast, we do not see an evident explanation for the stronger cold effect observed in the municipalities with a greater proportion of high-educated people and this should be further investigated. Our analysis also showed a higher heat effect in the most built-up municipalities with higher levels of non-vegetated surfaces, as well as a reduced number of cold-related deaths compared with less built-up areas. Evidence of higher heat effects and lower cold effects in urbanized areas has been reported elsewhere [15,50]. In an increasingly urbanized world, the urban population is expected to grow [51] and will thereby increasingly carry the heat burden associated with the urban heat island effect [52] even though the city can be seen to have a protective impact during winter.

Comparisons of the health impacts of temperature in different sub-populations contribute to the identification of vulnerable populations and risk factors to be addressed in the planning of public health interventions and prevention strategies. In Belgium, the cell Environment-Health, consisting of all Belgian administrations responsible for environment and/or health, has created the National Environment and Health Action Plan (NEHAP) as a framework to plan and implement actions to reduce the impact of environmental stressors on public health. This assembly coordinates the heatwave surveillance and warning system and the distribution of information on the health impact of extreme heat towards the general public, as well as towards health professionals.

This is the first multi-city study providing representative estimates of mortality due to low and high temperatures with information on cause of death and characteristics for different population groups in Belgium. Individual information such as the use of air conditioners that can modify adaptive mechanisms [53] or pre-existing conditions and chronic diseases which can interact in the relation between temperature and mortality [22,54,55] were, however, not included. The inclusion of 307,859 deaths provides good statistical power for an accurate description of temperature-related effects. We used temperatures estimated at the level of the municipality, a small geographical scale especially when studying urban municipalities. Nevertheless, we do not know the extent to which it correlates to micro-environmental temperatures. A variety of modeling options were explored, including the shape of the exposure–response curve and lag structure. We used state-of-the-art statistical methods, in particular, a multivariate meta-analysis with a distributed lag non-linear model to estimate and pool the non-linear and delayed relation between temperature and mortality [37,38]. Our analysis provides a comprehensive picture of the temperature–mortality association with a thorough investigation of the lag complexities. Modeling the cumulative effects of temperature on mortality including various lag periods is important to fully understand the impact of cold and heat. Here, we investigated the risk cumulated over lag times from the same day to up 7, 14, 21, and 28 days. These specific lag structures were intended to be representative and comparable with other studies [19,39,40] and not to reflect the only, or the exact, lag measurements appropriate for temperature–mortality studies.

## 5. Conclusions

The present study provides important knowledge for the planning of adequate public health interventions. Public health interventions should focus on the burden related to both low and high temperatures, with special attention to vulnerable populations who were identified in this study. Currently implemented mitigation measures might overlook the vulnerability of women and persons under 65 years old, and more attention should be given to urban areas with low vegetation cover. In the context of climate change, these findings have important implications for the proposal of adaptation actions and measures.

## Figures and Tables

**Table 1:** Summary statistics of population, mortality, and temperature by agglomeration in Belgium, 2010–2015.

Agglomeration	Municipalities	Deaths	Population ^1^	Daily Maximum Temperature (°C)							MMT ^2^	
	*n* (%)	*n* (%)	*n* (%)	Min	p_1_ ^3^	p_25_ ^3^	Median	p_75_ ^3^	p_99_ ^3^	Max	T (°C)	p ^3^
Antwerp	30 (13.8)	54,096 (17.6)	189,437 (18.3)	−6.9	−1.2	9.8	15.6	20.5	31.5	36.2	25.6	92.6
Brussels	62 (28.4)	89,070 (28.9)	306,146 (29.6)	−7.5	−1.6	9.5	15.6	20.5	31.3	36.2	23.6	87.5
Leuven	27 (12.4)	22,973 (7.5)	85,246 (8.2)	−9.7	−1.6	9.6	15.8	20.8	32.2	36.3	-	-
Bruges	10 (4.6)	16,142 (5.2)	65,432 (6.3)	−4.9	−0.5	9.8	14.9	19.7	29.4	35.7	-	-
Ghent	22 (10.1)	28,748 (9.3)	102,973 (10.0)	−5.4	−0.9	9.7	15.4	20.2	30.3	35.9	25.0	92.0
Charleroi	14 (6.4)	26,628 (8.6)	74,395 (7.2)	−7.3	−2.2	8.9	15.0	19.9	31.1	34.9	21.0	79.3
Mons	13 (6.0)	15,932 (5.2)	44,241 (4.3)	−5.6	−1.7	9.5	15.5	20.3	31.0	35.0	-	-
Liège	24 (11.0)	37,529 (12.2)	112,923 (10.9)	−8.8	−2.6	8.9	15.0	20.3	31.8	38.3	23.9	88.7
Namur	16 (7.3)	16,741 (5.4)	52,649 (5.1)	−8.3	−2.7	8.7	14.9	20.0	31.0	34.9	24.2	90.5
All	218	307,859	1,033,442	−9.7	−1.7	9.4	15.4	20.4	31.3	38.3	23.1	86.3

^1^ Population aged ≥65 years old on 1 January 2015. ^2^ MMT: agglomeration-specific and pooled overall minimum mortality temperatures with their corresponding percentiles (mortality–temperature association cumulated over lags of 0–21 days). ^3^ p: percentile of the temperature distribution.

**Table 2:** Cold effect (1st and 5th percentiles of temperature versus minimum-mortality temperature) and heat effect (95th and 99th percentiles of temperature versus minimum-mortality temperature) of temperature on mortality cumulated over lags of 0–21 days, 2010–2015.

		RR (95% CI) ^1^			
	Deaths	Cold Effect		Heat Effect	
	*n* (%)	p_1_ (−1.7 °C) ^2^	p_5_ (2.3 °C) ^2^	p_95_ (26.7 °C) ^2^	p_99_ (31.3 °C) ^2^
All	307,859 (100)	1.32 (1.21–1.44)	1.26 (1.19–1.34)	1.04 (1.01–1.07)	1.21 (1.08–1.36)
Causes of death					
Cardiovascular	91,327 (29.7)	1.26 (0.93–1.71)	1.31 (1.03–1.68)	1.01 (0.95–1.08)	1.14 (0.89–1.45)
IHD ^3^	26,132 (8.5)	1.46 (1.17–1.81)	1.42 (1.11–1.81)	1.05 (0.97–1.14)	1.45 (0.99–2.13)
Cerebrovascular	21,718 (7.1)	1.21 (0.89–1.63)	1.25 (1.08–1.44)	1.02 (0.97–1.07)	1.12 (0.76–1.64)
Respiratory	34,493 (11.2)	1.02 (0.74–1.40)	1.20 (1.02–1.41)	1.06 (0.94–1.19)	1.06 (0.69–1.63)
COPD ^4^	13,937 (4.5)	1.97 (1.29–3.01)	1.66 (1.31–2.12)	1.02 (0.81–1.28)	1.17 (0.50–2.76)
Other natural	182,039 (59.1)	1.29 (1.17–1.42)	1.21 (1.13–1.29)	1.03 (1.00–1.06)	1.18 (1.04–1.34)
Adjustment for air pollutants					
PM_2.5_, lag 1 day		1.34 (1.22–1.46)	1.27 (1.19–1.35)	1.04 (1.01–1.07)	1.21 (1.08–1.36)
PM_2.5_, lag 3 days		1.32 (1.21–1.45)	1.26 (1.19–1.34)	1.04 (1.01–1.07)	1.22 (1.09–1.36)
NO_2_, lag 1 day		1.34 (1.22–1.46)	1.27 (1.19–1.34)	1.04 (1.01–1.07)	1.21 (1.08–1.36)
NO_2_, lag 3 days		1.34 (1.22–1.47)	1.27 (1.19–1.35)	1.04 (1.01–1.07)	1.21 (1.09–1.36)
O_3_, lag 1 day		1.33 (1.22–1.45)	1.26 (1.19–1.34)	1.03 (1.01–1.06)	1.20 (1.08–1.34)
O_3_, lag 3 days		1.35 (1.24–1.47)	1.28 (1.21–1.35)	1.04 (1.00–1.07)	1.21 (1.07–1.36)

^1^ RR: relative risks and their 95% confidence intervals for temperature versus minimum mortality temperature (i.e., 23.1 °C). RR were calculated from the agglomeration-specific overall cumulative associations between temperature and mortality (lag of 0–21 days). ^2^ p: percentile of the temperature distribution and the corresponding temperature in Celsius degrees. ^3^ IHD: ischemic heart diseases. ^4^ COPD: chronic obstructive pulmonary diseases.

## Data Availability

Restrictions apply to the availability of these data. Data were obtained from the Belgian Statistical Office and are available from the authors with the permission of the Belgian Statistical Office.

## Supplementary Materials

The following supporting information can be downloaded at: https://www.mdpi.com/article/10.3390/ijerph19073763/s1, Figure S1: Agglomeration-specific and pooled overall temperature-mortality associations cumulated over lags of 0–7 days, 2010–2015; Figure S2: Agglomeration-specific and pooled overall temperature-mortality associations cumulated over lags of 0–14 days, 2010–2015; Figure S3: Agglomeration-specific and pooled overall temperature-mortality associations cumulated over lags of 0–28 days, 2010–2015; Figure S4: Pooled overall cumulative temperature-mortality associations by sex in people ≥85 years, cumulated over lags of 0–7 days, 2010–2015; Table S1: Cold effect (1st and 5th percentiles of temperature versus minimum-mortality temperature) and heat effect (95th and 99th percentiles of temperature versus minimum-mortality temperature) of temperature on mortality cumulated over lags of 0–21 days, 2010–2015; Table S2: Cold effect (1st and 5th percentiles of temperature versus minimum-mortality temperature) and heat effect (95th and 99th percentiles of temperature versus minimum-mortality temperature) of temperature on mortality cumulated over lags of 0–7, 0–14, 0–21 and 0–28 days, 2010–2015.
