# Supplementary material for: Impact of Short-Term Exposure to Extreme Temperatures on Mortality: A Multi-City Study in Belgium
Source: Int J Environ Res Public Health. 2022 Mar 22;19(7):3763. doi: 10.3390/ijerph19073763 (PMC8997565; doi:10.3390/ijerph19073763)
Supplement: Supplementary file 1 [file ijerph-19-03763-s001.zip › ijerph-1613281-supplementary.pdf]

Impact of short-term exposure to extreme temperatures on mortality: a multi-city study in Belgium

Supplementary Material

Table of Contents

TABLES 2

Table S1. Cold effect (1st and 5th percentiles of temperature versus minimum-mortality temperature) and heat effect (95th and 99th percentiles of temperature versus minimum-mortality temperature) of temperature on mortality cumulated over lags of 0–21 days, 2010-2015 2

Table S2. Cold effect (1st and 5th percentiles of temperature versus minimum-mortality temperature) and heat effect (95th and 99th percentiles of temperature versus minimum-mortality temperature) of temperature on mortality cumulated over lags of 0-7, 0-14, 0-21 and 0-28 days, 2010-2015 3

FIGURES 4

Figure S1. Agglomeration-specific and pooled overall temperature-mortality associations cumulated over lags of 0-7 days, 2010-2015 4

Figure S2. Agglomeration-specific and pooled overall temperature-mortality associations cumulated over lags of 0-14 days, 2010-2015 5

Figure S3. Agglomeration-specific and pooled overall temperature-mortality associations cumulated over lags of 0-28 days, 2010-2015 6

Figure S4. Pooled overall cumulative temperature-mortality associations by sex in people ≥ 85 years, cumulated over lags of 0-7 days, 2010-2015 7

Tables

Table S1. Cold effect (1st and 5th percentiles of temperature versus minimum-mortality temperature) and heat effect (95th and 99th percentiles of temperature versus minimum-mortality temperature) of temperature on mortality cumulated over lags of 0–21 days, 2010-2015

		RR (95% CI) [a]			
	Deaths	Cold effect		Heat effect	
	n (%)	p_1 (-1.7°C) [b]	p_5 (2.3°C) [b]	p_{95} (26.7°C) [b]	p_{99} (31.3°C) [b]
Age					
< 65 years	44,024 (14.3)	1.10 (0.88-1.36)	1.16 (0.96-1.39)	1.11 (1.05-1.17)	1.46 (1.19-1.78)
65-74 years	45,166 (14.7)	1.12 (0.80-1.57)	1.15 (0.99-1.33)	1.01 (0.94-1.08)	1.07 (0.75-1.51)
75-84 year	92,248 (30.0)	1.34 (1.16-1.54)	1.26 (1.17-1.35)	1.03 (0.99-1.07)	1.15 (0.97-1.36)
≥ 85 years	126,421 (41.1)	1.44 (1.25-1.65)	1.33 (1.22-1.46)	1.01 (0.96-1.07)	1.15 (0.90-1.48)
Sex					
Male	146,095 (47.5)	1.30 (1.18-1.44)	1.21 (1.14-1.28)	1.02 (0.98-1.05)	1.10 (0.92-1.31)
Female	161,764 (52.5)	1.32 (1.16-1.49)	1.30 (1.19-1.42)	1.05 (1.01-1.10)	1.29 (1.11-1.50)
Education					
low	87,502 (28.4)	1.21 (1.01-1.46)	1.28 (1.14-1.44)	1.02 (0.98-1.07)	1.21 (0.99-1.47)
high	69,069 (22.4)	1.55 (1.16-2.07)	1.37 (1.11-1.69)	1.02 (0.94-1.10)	1.24 (0.99-1.55)
% built-up area					
low	71,950 (23.4)	1.51 (1.27-1.79)	1.32 (1.19-1.47)	1.03 (0.95-1.11)	1.23 (0.92-1.65)
high	81,140 (26.4)	1.28 (1.05-1.55)	1.19 (1.05-1.34)	1.07 (1.02-1.13)	1.41 (1.17-1.70)
Municipality of death=residence	192,912 (62.7)	1.28 (1.12-1.46)	1.24 (1.15-1.33)	1.03 (0.99-1.07)	1.15 (0.99-1.34)
Adjustment for humidity					
	lag 1 day	1.34 (1.23-1.47)	1.27 (1.19-1.35)	1.04 (1.01-1.07)	1.22 (1.08-1.36)
	lag 3 days	1.37 (1.26-1.49)	1.29 (1.22-1.36)	1.03 (1.00-1.06)	1.21 (1.08-1.35)

[a] RR: relative risks and their 95% confidence intervals for temperature versus minimum mortality temperature (i.e. 23.1 °C). RR were calculated from the agglomeration-specific overall cumulative associations between temperature and mortality (lag of 0-21 days)

[b] p: percentile of the temperature distribution and the corresponding temperature in Celsius degrees

Table S2. Cold effect (1st and 5th percentiles of temperature versus minimum-mortality temperature) and heat effect (95th and 99th percentiles of temperature versus minimum-mortality temperature) of temperature on mortality cumulated over lags of 0-7, 0-14, 0-21 and 0-28 days, 2010-2015

					RR [a] (95% CI)			
	k [b]		MMT [c]		Cold effect		Heat effect	
Lag period	t	l	T (°C)	p [d]	p_1 (-1.7°C) [d]	p_5 (2.3°C) [d]	p_{95} (26.7°C) [d]	p_{99} (31.3°C) [d]
Main analysis								
0-7 days	3	1	21.0	78.0	1.05 (1.01-1.09)	1.07 (1.03-1.11)	1.08 (1.06-1.11)	1.33 (1.24-1.43)
0-14 days	3	2	22.1	82.5	1.17 (1.11-1.24)	1.16 (1.11-1.21)	1.06 (1.04-1.09)	1.29 (1.19-1.41)
0-21 days	3	2	23.1	86.3	1.32 (1.21-1.44)	1.26 (1.19-1.34)	1.04 (1.01-1.07)	1.21 (1.08-1.36)
0-28 days	3	2	23.9	88.8	1.47 (1.32-1.63)	1.33 (1.24-1.43)	1.02 (0.99-1.06)	1.18 (1.03-1.35)
Sensitivity analysis								
0-7 days	5	3	21.6	80.5	1.05 (1.01-1.10)	1.10 (1.05-1.15)	1.07 (1.02-1.12)	1.33 (1.24-1.42)
0-14 days	5	4	23.1	86.3	1.16 (1.09-1.24)	1.22 (1.15-1.30)	1.04 (0.99-1.09)	1.31 (1.20-1.42)
0-21 days	5	4	25.7	93.2	1.29 (1.16-1.43)	1.33 (1.23-1.43)	1.01 (0.98-1.03)	1.26 (1.11-1.44)
0-28 days	5	4	25.9	93.6	1.44 (1.25-1.65)	1.43 (1.30-1.57)	1.00 (0.98-1.02)	1.27 (1.07-1.52)

[a] RR: relative risks and their 95% confidence intervals for temperature versus minimum mortality temperature. RR were calculated from the agglomeration-specific overall cumulative associations between temperature and mortality

[b] k: number of internal knots for the temperature (t) and lag (l) dimensions of the crossbasis function in the distributed lag non-linear models

[c] MMT: overall and agglomeration, specific minimum mortality temperatures with their corresponding percentiles

[d] p: percentile of the temperature distribution and corresponding temperature in Celsius degrees T (°C)

Figures

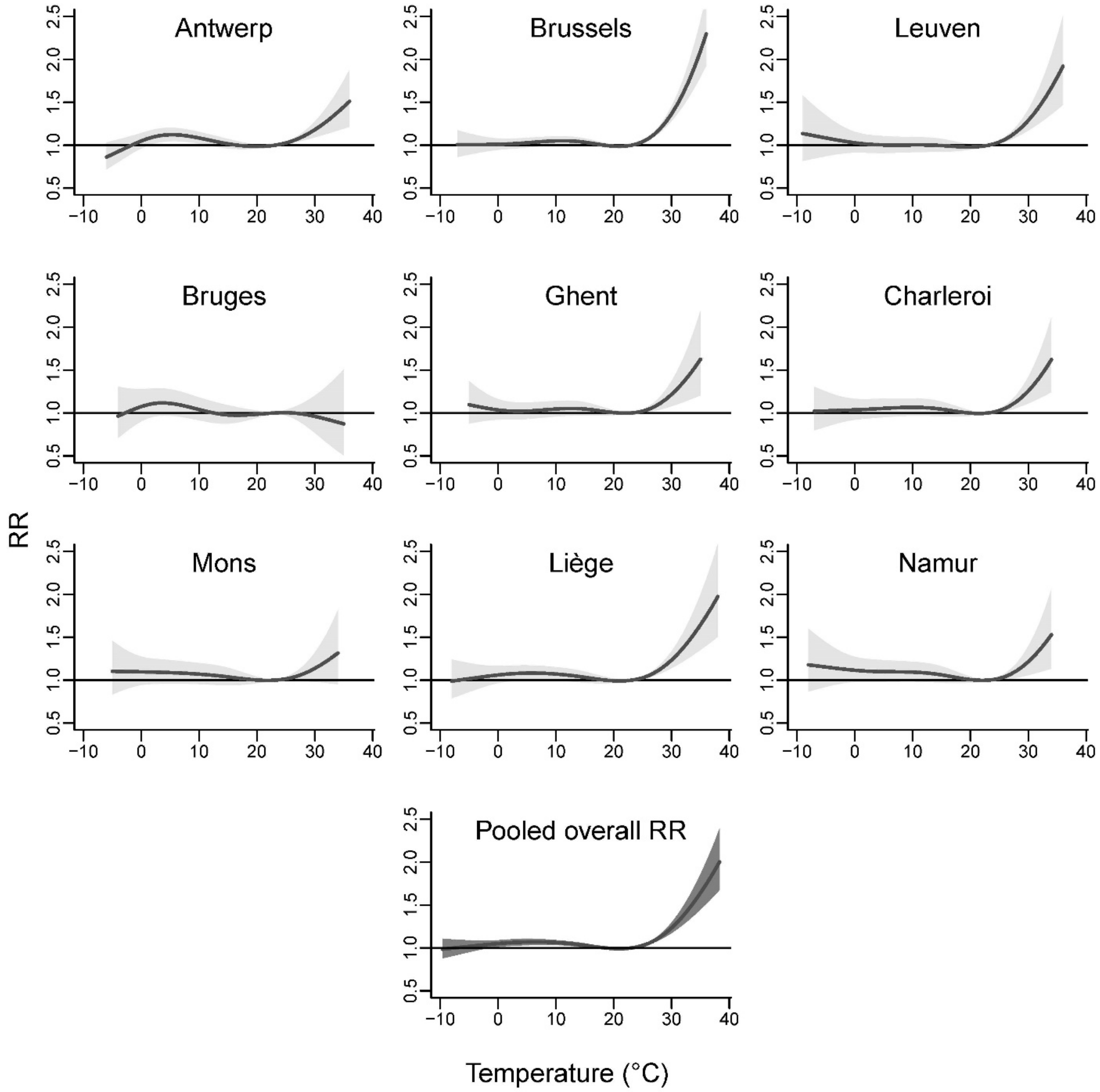

Figure S1. Agglomeration-specific and pooled overall temperature-mortality associations cumulated over lags of 0-7 days, 2010-2015

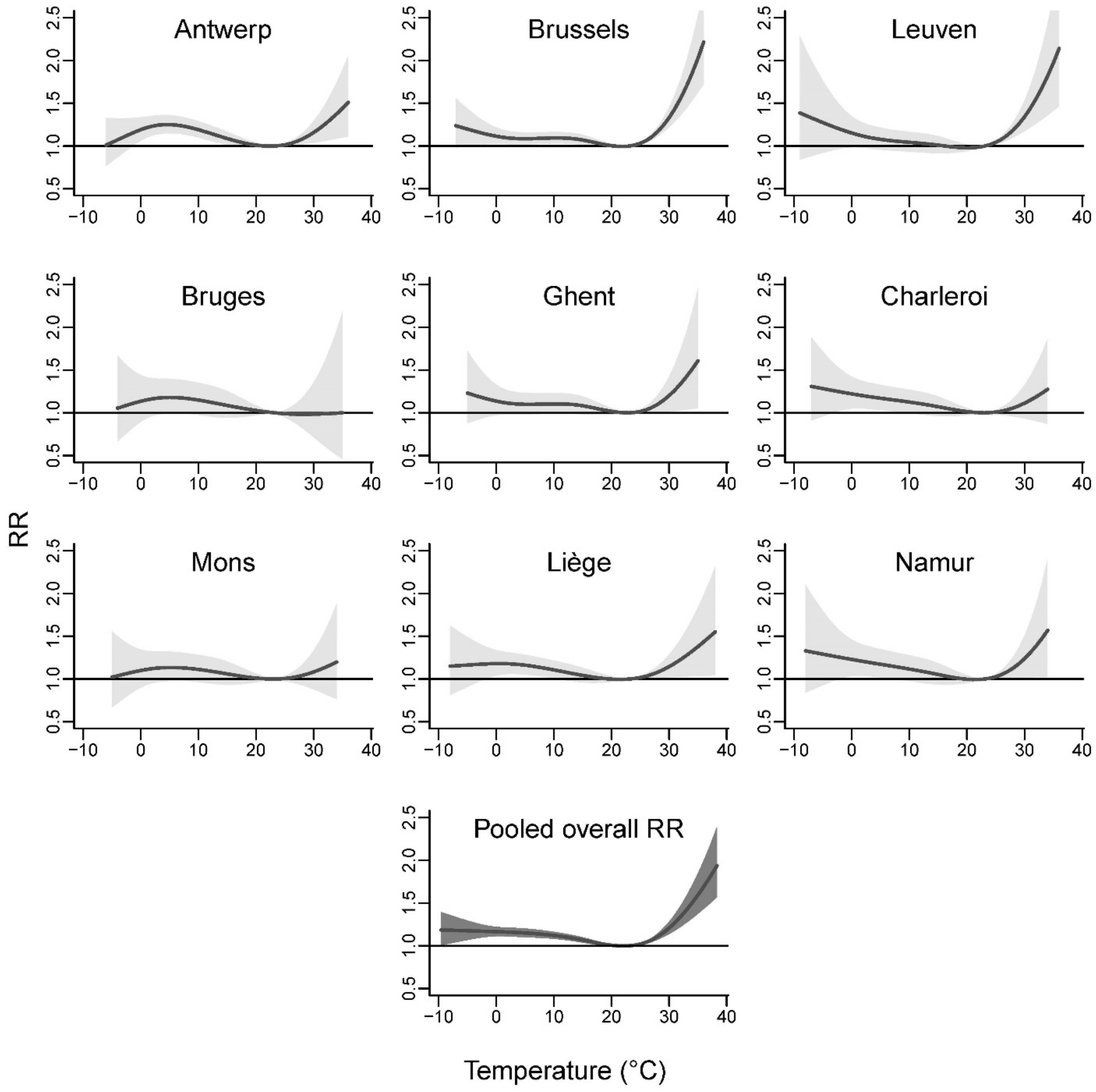

Figure S2. Agglomeration-specific and pooled overall temperature-mortality associations cumulated over lags of 0-14 days, 2010-2015

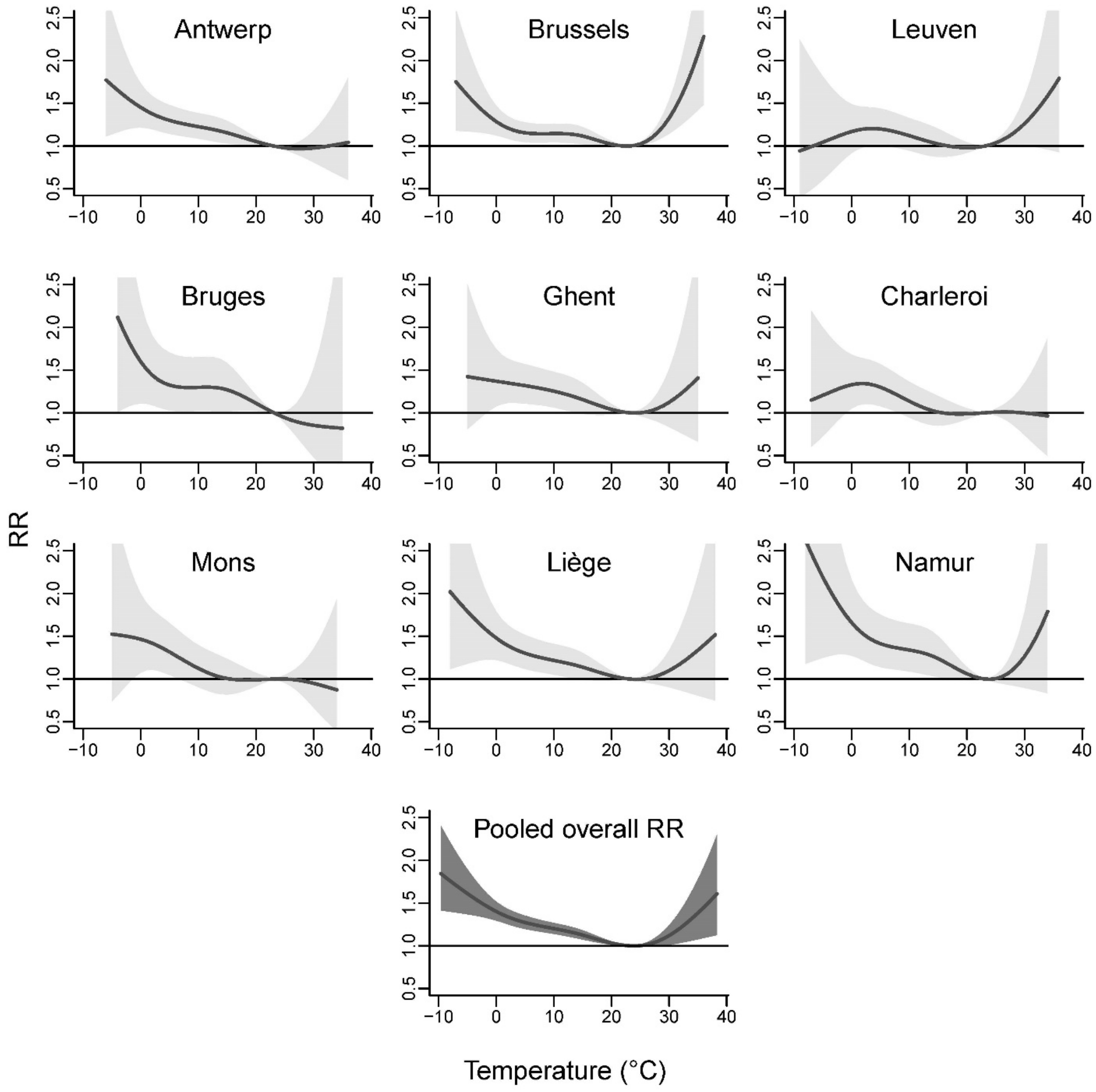

Figure S3. Agglomeration-specific and pooled overall temperature-mortality associations cumulated over lags of 0-28 days, 2010-2015

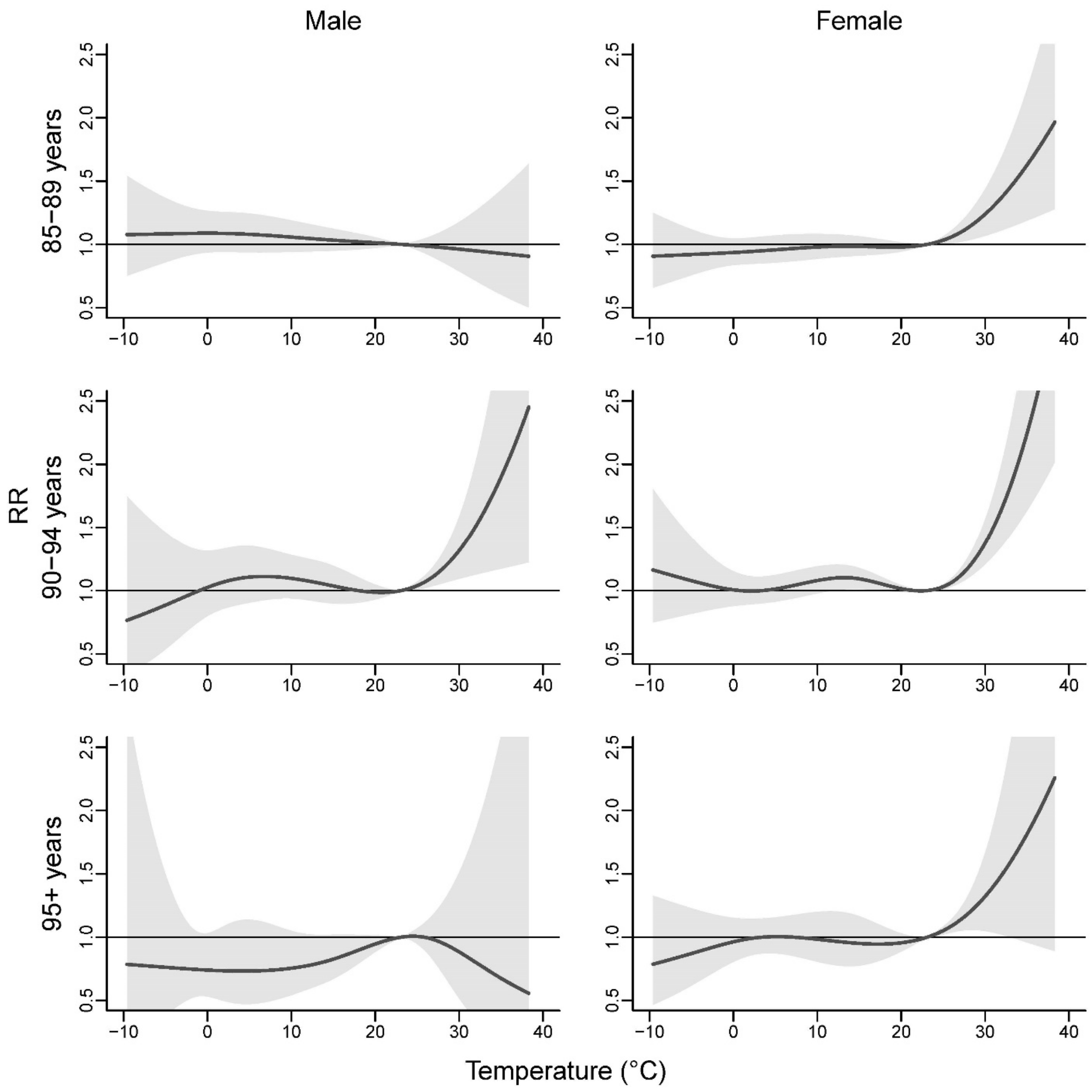

Figure S4. Pooled overall cumulative temperature-mortality associations by sex in people ≥ 85 years, cumulated over lags of 0-7 days, 2010-2015
